# Relation of Mitral Annulus and Left Atrial Dysfunction to the Severity of Functional Mitral Regurgitation in Patients with Dilated Cardiomyopathy

**DOI:** 10.1155/2020/3261714

**Published:** 2020-07-09

**Authors:** Sorina Mihaila Baldea, Denisa Muraru, Marcelo Haertel Miglioranza, Sabino Iliceto, Dragos Vinereanu, Luigi Paolo Badano

**Affiliations:** ^1^University of Medicine and Pharmacy Carol Davila, Bucharest, Romania; ^2^Istituto Auxologico Italiano IRCCS, Department of Cardiovascular Neural and Metabolic Sciences, San Luca Hospital, Milan, Italy; ^3^University of Milano-Bicocca, Department of Medicine and Surgery, Milan, Italy; ^4^Cardiology Institute of Rio Grande do Sul, Porto Alegre, Brazil; ^5^University of Padua, Padua, Italy

## Abstract

**Methods:**

56 patients (58 ± 17 years, 42 men) with DCM and FMR and 52 controls, prospectively enrolled, underwent 3DTTE dedicated for mitral valve (MV), LA, and left ventricle (LV) quantitative analysis.

**Results:**

Patients with FMR vs. controls presented increased MA size and sphericity during the entire systole, whereas MA fractional area change (MAFAC) and MA displacement were decreased (15 ± 5 vs. 28 ± 5%; and 5 ± 3 vs. 10 ± 2 mm, *p* < 0.001). In patients with moderate/severe FMR, MA diameters correlated with PISA radius, EROA, and regurgitant volume (Rvol), as also did the MA area (with PISA radius, EROA, and Rvol: *r* = 0.48, *r* = 0.58, and *r* = 0.47, *p* < 0.05). MAFAC correlated inversely with EROA and Rvol (*r* = −0.32 and *r* = −0.35, *p* < 0.05), with both active and total LA emptying fractions and with LV ejection fraction as well. In a stepwise multivariate regression model, decreased MAFAC and increased LA volume independently predicted patients with severe FMR.

**Conclusions:**

Patients with DCM and FMR have MA geometry remodeling and contractile dysfunction, correlated with the severity of FMR. MA contractile dysfunction correlated with both LA and left LV pumps dysfunctions and predicted patients with severe FMR. Our results provide new insights that might help with better selection of patients for MV transcatheter procedures.

## 1. Introduction

Functional mitral regurgitation (FMR) is a common complication in patients with ischemic or nonischemic dilated cardiomyopathy (DCM), increasing mortality, and the risk of rehospitalization [1]. In patients with FMR, surgical mitral valve (MV) treatment showed suboptimal outcomes in terms of morbidity and mortality [2, 3], while interventional treatment provided controversial results [4, 5], suggesting that selection of patients is crucial for treatment effectiveness. Thus, in order to select better the patients for the transcatheter mitral valve procedures, assessment of MV geometry should be mandatory [6].

Recent studies suggested an association between the anteroposterior diameter of the mitral annulus (MA) and FMR recurrence after MV interventional treatment [7]. However, the MA is a complex tridimensional structure, which cannot be characterized by a single annular diameter [8, 9]. Therefore, understanding the geometrical and functional changes occurring in the MA, as well as their relations with the LA and LV dilation and function, is essential in patients with DCM and FMR [10]. This is now possible with the progress of three-dimensional (3D) full-volume acquisition with high temporal and spatial resolution using transthoracic echocardiography (3DTTE), coupled with the development of specific software packages, which enable a detailed quantitative analysis of MA [11], LV, and LA geometry and functions.

Accordingly, we designed our study (i) to characterize MA geometry and dynamics changes, as the remodeling of the MA, by using 3DTTE in patients with DCM and FMR, in comparison with healthy controls; (ii) to assess the relationship between MA remodeling and severity of FMR; and (iii) to assess the relationship between MA dysfunction and severity of LV and LA dilation and dysfunction.

## 2. Methods

### 2.1. Study Population

We conducted a prospective study, in which we enrolled 56 patients with mild-to-severe FMR due to ischemic and nonischemic DCM and compared them with 52 control subjects, similar in age, gender, and BSA. All subjects were in sinus rhythm and had complete 3DTTE datasets for MV, LA, and LV. Patients with more than mild aortic, pulmonic, or tricuspid valve disease, significant MV calcifications, or poor-quality image of the 3DE datasets were excluded. Control subjects were healthy volunteers free of any cardiovascular history or risk factors. Heart rate, blood pressure, height, and weight were measured in all subjects immediately before the echocardiographic examination. Body surface area (BSA) was calculated [12]. Etiology of DCM, NYHA class, and the presence of wide QRS (>120 msec) on ECG were recorded. The Local Ethics Committee approved the study, and all subjects provided an informed consent.

### 2.2. Echocardiography

Echocardiography was performed in accordance with a standardized acquisition protocol, with a Vivid E9 system (GE Vingmed Ultrasound, Horten, Norway), equipped with M5S and 4V probes. Standard transthoracic echocardiography was performed to assess LV size and function and etiology and severity of FMR in patients with DCM. Then, to obtain high temporal resolution of the datasets, separate 3D full-volume multibeat acquisitions of the MV, LV, and LA were obtained by combining six consecutive ECG-triggered subvolumes, during breath-hold and avoidance of patient or probe movement. Care was taken to encompass the MV in the full volume throughout the cardiac cycle and the entire LV and LA in the dedicated dataset. By using multislice display, the absence of stitching artifacts in the acquired dataset was carefully checked.

### 2.3. Image Analysis

The severity of FMR was graded as trivial/mild, moderate, and severe, using two-dimensional (2D) echocardiography, according to the current guidelines [13]. Qualitative, semiquantitative, and quantitative parameters were used, and 2D biplane vena contracta, 2D PISA radius (PISArad), effective regurgitant orifice area (EROA), and regurgitant volume (RVol) were measured/calculated [13]. In patients with trivial/mild MR by colour Doppler, 2D PISArad, RVol, and EROA were null and excluded from the statistical analysis.

**Figure 1:**
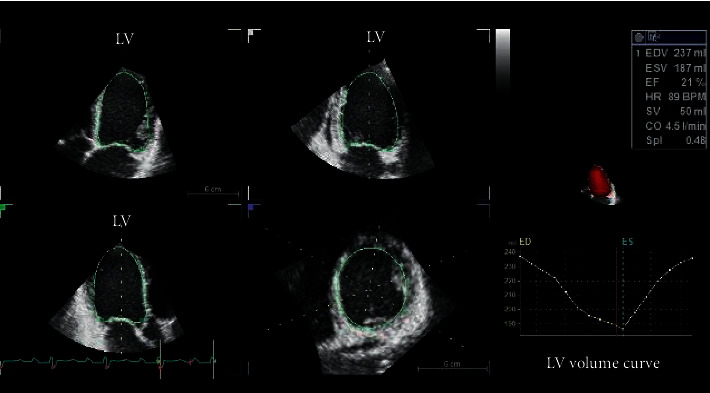
Left ventricular quantitative analysis using 3D transthoracic echocardiography in a patient with dilated cardiomyopathy and functional mitral regurgitation. LV: left ventricle.

LV 3DTTE datasets were stored digitally in raw-data format for offline analysis (Auto-LVQ, EchoPAC BT12, GE Vingmed Ultrasound, Horten, Norway), as previously described and validated against cardiac magnetic resonance (CMR) [14, 15] (Figure 1).

**Figure 2:**
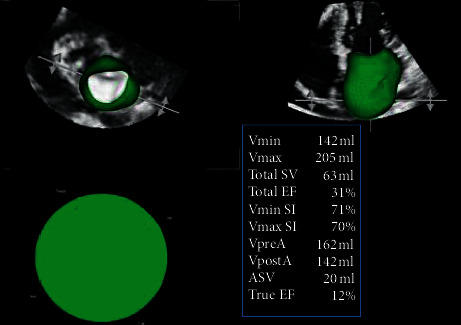
Left atrial quantitative analysis using 3D transthoracic echocardiography in a patient with dilated cardiomyopathy and functional mitral regurgitation.

LA 3DTTE datasets were converted to DICOM format and analyzed using dedicated software package designed for volumetric analysis of the LA and recently validated against CMR (LA analysis 2.3, TOMTEC Imaging Systems, Unterschleissheim, Germany) [16]. At the beginning of the 3D analysis workflow, LA datasets were automatically sliced in 4-chamber, 2-chamber, and long-axis apical planes and a short-axis plane. Translating and rotating 4-chamber plane to obtain orthogonal nonforeshortened planes of the LA in all 3 apical views allowed rapid manual data set alignment. In each apical view, LA blood-tissue interface was manually initialized on 2 frames, identifying maximum and minimum LA volumes (LAVmax and LAVmin). A 3D surface of LA volume was then generated for each frame throughout the cardiac cycle resulting in a dynamic cast of the LA cavity. For each consecutive frame, the voxel count inside the 3D LA surface was used to measure LA volume, resulting in a smooth interpolated time–volume curve from which LAVmax, LAVmin, and preatrial contraction LA volume (LAVpreA) were obtained [17]. From LA volumes, the software automatically calculated total, passive, and active LA emptying fractions (LAEFs) [16] (Figure 2).

**Figure 3:**
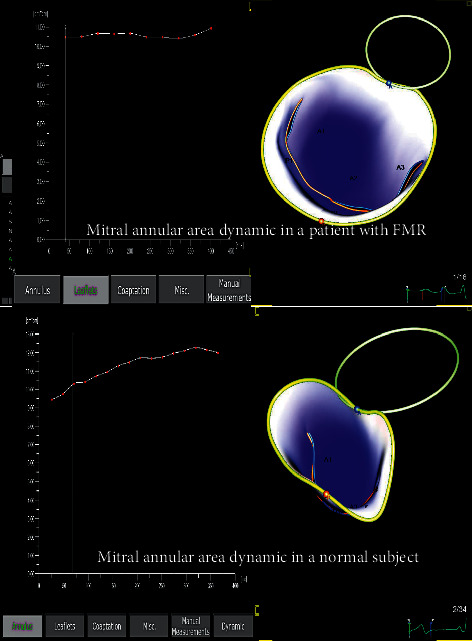
Mitral annular area change by 3D transthoracic echocardiography throughout the cardiac systole, in a patient with functional mitral regurgitation (upper panel) and a normal subject (lower panel); mitral annulus from the patient with functional mitral regurgitation is dilated, spherical and shows smaller changes in the annular area during the cardiac systole than the normal mitral annulus.

MV 3DTTE datasets were converted to DICOM format and analyzed using dedicated software for MV analysis (TOMTEC, 4D-MV assessment 2.3, Unterschleissheim, Germany), as previously described [18]. Briefly, MA measurements were performed in early-systole—the frame after the MV closure (MVC); end-systole—the frame just before MV starts to open; and in mid-systole–the frame midway between MVC and end-systole. After adding anatomical landmarks, the software created a 3D model of the MA and MV leaflets, first at mid-systole (*static analysis*) and then during the cardiac systole (*dynamic analysis*) (Figure 3). MA measured parameters were 3D and 2D (projected) areas and circumference; anteroposterior (AP) and anterolateral-posteromedial (ALPM) diameters; sphericity index; height and nonplanarity angle to quantify its “saddle shape”; and Ao-AP angle, as the angle between the aorta and MA plane. In addition, MV commissural diameter, anterior leaflet area and length (ALA and ALL), posterior leaflet area and length (PLA and PLL), tenting height, area, and volume were also measured. For all parameters, values at MVC, mid-systole, and end-systole, their minimal value, and the time interval from MVC to moment of their minimal value were recorded and expressed as percentage (%) of the total duration of the systole [19]. MA diameters, area, and circumference were normalized to BSA. The software package also provided MA displacement, displacement velocity, and MA area fractional change during systole. The fractional change (difference between the maximal and minimal value divided by the maximal value and expressed as percentage) of the MA circumference, AP diameter, and ALPM diameter were also calculated [19].

### 2.4. Statistical Analysis

Normal distribution of variables was checked by Kolmogorov–Smirnov test. Continuous variables were summarized as mean ± SD, and categorical variables were reported as percentage. Variables were compared between groups using unpaired *t*-test. Pearson's correlation was used to analyze the relationships between MR severity parameters and MA function and between MA function and LA and LV parameters. Multiple linear backward regression tests were used to identify the parameters that independently correlated with severe MR. All analyses were performed using SPSS version 20.0 (SPSS, Inc., Chicago, USA) and MedCalc version 10.0.1.0 (MedCalc Software, Mariakerke, Belgium). Differences among variables were considered significant at *p* < 0.05. Our group has previously reported the reproducibility for the echocardiographic methods, in unselected patients with MV disease [18].

## 3. Results

### 3.1. General Characteristics

The general characteristics of the study population are summarized in Table 1. Age, gender, blood pressure, and BSA were similar between groups. As expected, patients with FMR had larger LV volumes and lower LVEF than controls. Patients with DCM had an ischemic etiology in 40 cases (71%); they were in NYHA functional class I in 5 cases (9%), II in 26 cases (46%), and III in 25 cases (45%). 17 patients (30%) had mild, 25 (45%) moderate, and 14 (25%) severe FMR. PISArad was 6±3 mm, available in 42 patients (75%), while EROA was 0.17 ± 0.12 cm^2^ and RVol 26 ± 19 ml, available in 33 patients (59%). Temporal resolution of the 3D datasets for MA analysis was 32 ± 7 volume/sec in patients with FMR and 35 ± 6 volume/sec in control subjects (*p*=0.87). The number of systolic frames in each dataset ranged from 9 to 17, depending on heart rate or acquisition settings. Static analysis of the MA was feasible in all subjects. Dynamic analysis of MA was feasible in 54 patients with FMR (96%) and in 48 control subjects (92%).

### 3.2. Dimensions and Geometry of the MA in Patients with FMR vs. Controls

**Figure 4:**
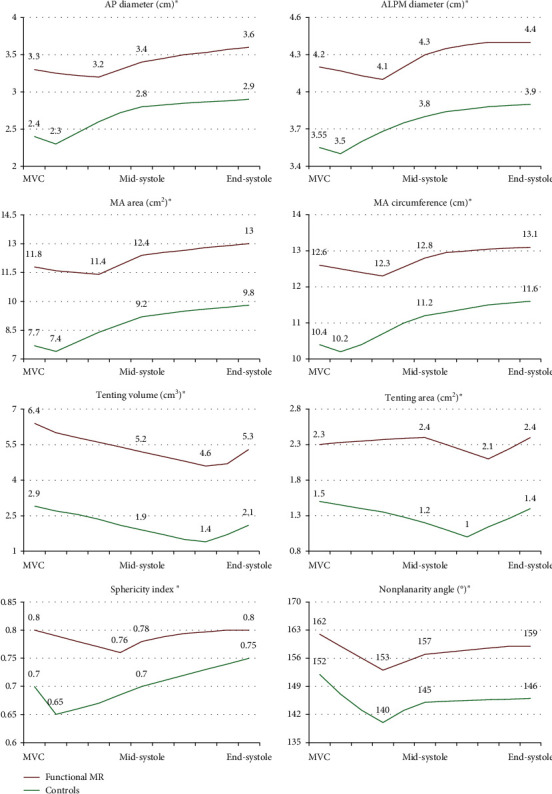
Dynamic changes of the mitral annulus geometry parameters during the cardiac systole in patients with functional mitral regurgitation (in red) and in control subjects (in green). AP: anteroposterior; ALPM: anterolateral-posteromedial; MA: mitral annulus; MVC: mitral valve closure. ^∗^Statistically significant at every analyzed reference frame, *p* < 0.001.

Mitral annulus static analysis is shown in Table 2. Patients with FMR, when compared with controls, had larger MA dimensions. They had 35% larger MA area, by 3DTTE. Moreover, they had larger ALA and PLA, but with a different extent of MV leaflet enlargement vs. controls, resulting in a lower ALA/PLA ratio. Meanwhile, patients with FMR vs. controls had increased MA sphericity and nonplanarity angle, but similar MA height. They had increased MV tenting volume, area, and height, while Ao-AP angle was wider. Mitral annulus dynamic analysis is presented in Table 3, while dynamic graphic of MA dimensions and geometry is displayed in Figure 4. Patients with FMR, when compared with controls, had larger MA dimensions at every reference frame of the cardiac systole. They had increased MA sphericity index, due to unequal increase in MV leaflets areas, and increased MV tenting volume, area, and height, throughout the entire systole. Meanwhile, they had decreased MA height and wider MA nonplanarity angle and MA Ao-AP angle than controls. Patients with FMR presented prolonged times to occurrence of minimum AP, ALPM, and commissural diameters and to occurrence of minimal MA area and circumference. They also had prolonged times to occurrence of minimal MA sphericity, height, and minimal Ao-AP angle. Patients with FMR presented decreased fractional changes of the MA parameters than controls (Table 4).

### 3.3. MA Remodeling and Dysfunction and Severity of FMR

Comparisons between LV, LA, and MA parameters in patients with trivial/mild, moderate, and severe FMR are shown in Table 5. Patients with moderate FMR had increased indexed LVEDV, lower LVEF, but similar LAVmax, than patients with trivial/mild FMR; meanwhile, they had increased MA dimensions and decreased MA area fractional change and MA displacement. Patients with severe MR had similar volumes and LVEF compared with patients with moderate MR, but increased LAVmax and MA dimensions, and decreased MA area fractional change.

In patients with moderate and severe FMR, MA diameters (AP and ALPM) correlated with all quantitative parameters of FMR severity (with PISArad: *r* = 0.56 and *r* = 0.43; with EROA: *r* = 0.66 and *r* = 0.45; and with RVol: *r* = 0.53 and *r* = 0.39, all *p* < 0.05), as did also MA area (with PISArad, EROA, and Rvol *r* = 0.48, *r* = 0.58, and *r* = 0.47, respectively, all *p* < 0.05). MV tenting volume correlated with PISArad (*r* = 0.41, *p* < 0.01) and with Rvol (*r* = 0.38, *p* < 0.05), while MV tenting correlated only with PISArad (*r* = 0.34, *p* < 0.05). MA fractional area change correlated inversely with EROA and Rvol (*r* = −0.32 and *r* = −0.35, both *p* < 0.05).

### 3.4. Independent Predictors for Severe MR

In a multiple backward linear regression model using indexed LVEDV; LAVmax; MA area; LVEF; active and total LAEFs; AP diameter fractional change; and MA fractional area change, independent predictors for severe MR were the LAVmax (*p*=0.03), AP diameter fractional change (*p*=0.02), and MA fractional area change (*p*=0.003), respectively.

### 3.5. MA Dysfunction and the Severity of LV and LA Remodeling

**Figure 5:**
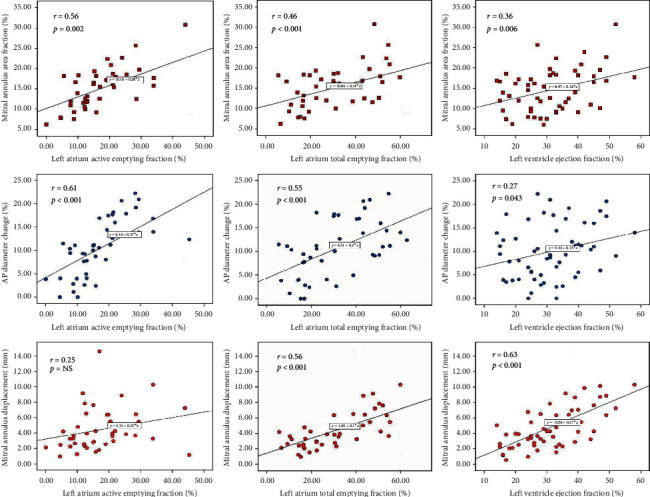
Relation between mitral annular functions and left atrial and left ventricular functions in patients with functional mitral regurgitation. AP: anteroposterior diameter.

MA area showed similar correlations with LVEDV and LAVmax (*r* = 0.55 and *r* = 0.54, both *p* < 0.001). MA area fractional change correlated with both active and total LAEFs, as well as with LVEF, while MA AP diameter fractional change showed similar behavior; MA displacement correlated only with LVEF (Figure 5).

## 4. Discussion

Our study showed that in patients with FMR there is geometric and functional remodeling of the MV, that can be summarized as follows: (i) increased MA size and sphericity, during the entire cardiac systole, was associated with a concomitant remodeling of the MV leaflets; (ii) MA was flatter, with increased MV tenting height, area, and volume during the cardiac systole; (iii) maximum MA contraction was not only decreased in amplitude, but also delayed in time; (iv) MA size and contractile dysfunction correlated with FMR severity; and (v) MA contractile dysfunction correlated with both LA and LV dysfunctions.

### 4.1. Mitral Annulus Geometry and Dynamics in Patients with Functional Mitral Regurgitation

MA remodeling and dysfunction have been reported as mechanisms of both ischemic and nonischemic FMR [10, 20, 21]. In ischemic MR, MA dysfunction was related to local LV remodeling [22] and severity of regurgitation [20]. MA remodeling and dysfunction are more prominent in patients with LV dysfunction and FMR than in those without FMR [10], despite the fact that isolated MA dilation failed to explain the occurrence of MR in patients without dilated LV [23]. Meanwhile, LA remodeling was suggested to have a role in ischemic MR through an atriogenic mechanism that determines MV leaflet tethering [24]. However, the relationship between MA dysfunction and LV and LA remodeling and its relation to MR severity has been explored insufficiently in patients with FMR.

Patients with mild-to-severe FMR showed MA dilation throughout the entire systole, as previously reported [10, 21]. However, our data are in contrast with those reported by Topilsky et al. [10], since our patients with FMR presented an increase of both AP and ALPM diameters of the MA and not of the AP diameter only. Still, an unequal increase in AP and ALMP size was noted in our FMR patients, which lead to a more spherical MA than in control subjects. In patients with moderate-to-severe FMR, the increase in AP and ALPM diameters was correlated with FMR severity. On the other hand, our data, showing the direct relation between MA size and shape and FMR severity, confirm and expand the data reported by Topilsky et al. [10], who showed that patients with LV dysfunction and FMR have larger AP size of the MA than patients with LV dysfunction without FMR. Moreover, although it is widely accepted that FMR is mainly due to regional and global remodeling of the LV, while the MV apparatus is normal, a study by Debonnaire et al. [25] contradicted the paradigm of the “normal mitral valve” and suggested a true remodeling of the entire MV leaflets occurring in patients with FMR. Our study showed the increase of both anterior and posterior MV leaflets areas, from the MA to the point of their coaptation, in patients with FMR. The software package that we used for the study did not measure the entire leaflet area, as in the study by Debonnaire et al., but “an effective” leaflet area, from the base to the coaptation point of the MV. However, it seems that the increase in this “effective leaflet's size” is not symmetric, since the ratio between ALA and PLA became almost unitary in our patients with FMR. In a recent paper published by Stolfo et al. [7], the increase in the AP diameter of the MA was associated with a higher recurrence of MR after percutaneous repair of functional MR, in patients with advanced heart failure. Our data suggest that further studies should be performed, in order to better select patients for percutaneous repair of the MV, based on MA parameters, as well.

MA was flatter in patients with FMR than in control subjects. Similar with the data reported by Topilsky et al. [10], our patients with FMR showed higher MV tenting volume, area, and height throughout the systole, but only MV tenting volume showed significant correlations with MR severity. Previous reports have shown that MV tenting volume was a significant predictor of MR severity [26] and of recurrence of MR after MV annuloplasty.

Beside MA enlargement and remodeling, patients with FMR also showed altered relation of the MA with the surrounding structures, presenting wider Ao-AP angle than control subjects. This has to be taken into account for annuloplasty techniques, as the angle Ao-AP proved to predict the occurrence of systolic anterior movement of the anterior mitral leaflet after MV annuloplasty [27]. Moreover, LV outflow obstruction is a potential complication of the new transcatheter mitral valve replacement procedure (MitraClip) that can be predicted during procedure planning since it is related not only to prosthesis design, but also to anatomy (septal thickness, LV size, Ao-AP angle, and anterior septal length) [28].

LV dyssynchrony has been reported as a mechanism of FMR, while cardiac resynchronization therapy (CRT) can reduce early systolic FMR in selected patients [29–31], but the detailed mechanism remains to be clarified. Delay in contraction of the MA, showed by us, might have a role in the early MV regurgitation during cardiac systole. Thus, future studies might address how CRT affects the timing of MA contraction.

### 4.2. Relation between MA Geometry and Function and LA and LV Size and Function in Patients with FMR

MA area fractional change was increasingly impaired in patients with FMR. We showed that the “contractile dysfunction” of the MA was associated with both LA and LV dysfunctions. In patients with DCM, Kwan et al. [32] reported that the extent of the decreased contraction of MA area and AP diameter was related to the extent of LV dysfunction, in terms of LVEF. In addition, previous experimental models showed the relation between MA contraction and LA function [33], and this has been reported in normal individuals too, using 2D echocardiography [34]. Several groups [35–37] suggested that, in patients with atrial fibrillation, LA remodeling may play a role in the pathogenesis of FMR through a new mechanism, the “atriogenic leaflet tethering.” Our results showed that in patients with dilated and dysfunctional LV, the dilation and dysfunction of the LA have an important role in the contractile dysfunction of the MA, beyond the role played by the LV dysfunction, and it is closely related to the severity of FMR. MA dysfunction, associated with LA remodeling, might be an additional “atriogenic” mechanism determining the worsening FMR from moderate to severe in patients with DCM, who had similar LV volumes and LVEF.

### 4.3. Predictors for Severe MR

Otsuji et al. [23] showed that MA dilation fails to solely explain MR severity in patients with atrial fibrillation vs. patients with DCM and FMR. Our study confirms that neither the MA enlargement nor the LV dilation is a predictor for MR severity. Conversely, from all parameters measured for the LV, LA, and MA size and functions, only the LA maximal volume and the contraction of AP diameter and of MA area are able to predict severe MR. Therefore, LA dilation associated with a decreased “contraction” of the MA might be an additional “atriogenic” mechanism that increases MR severity in patients with DCM, independently from the remodeling of the LV.

### 4.4. Limits of the Study

In our study, we did not assess the complex relationship between papillary muscle position, orientation, and FMR severity; however, this has been extensively assessed in previous studies [38–40]. The software package, used to assess MA dynamics, performs MA measurements only during the systolic phase, and not during the entire cardiac cycle. However, it has been shown that, even in normal subjects, the most important conformational changes of MA occur during the systolic phase of the cardiac cycle and less in the diastolic phase [41]. Moreover, we aimed to assess the MA dynamics, in parallel with the occurrence of the MR, during the systolic phase of the cardiac cycle.

## 5. Conclusions

Patients with dilated cardiomyopathy and functional mitral regurgitation have mitral annulus geometry remodeling and contractile dysfunction, which correlated with the severity of mitral regurgitation. Mitral annular contractile dysfunction correlated with both left atrial and left ventricular pump dysfunctions. Thus, these results enhance the association of atrial remodeling and dysfunction with the severity of functional mitral regurgitation severity, mediated through the mitral annular dysfunction. Our results may ensure further studies to use parameters of the mitral annular size and geometry, in order to help us to better select patients for transcatheter procedures, targeting the mitral annulus or the mitral valve.

## Figures and Tables

**Table 1:** General characteristics of patients with functional mitral regurgitation and control subjects.

	Functional mitral regurgitation	Control subjects	*p*
	(*N* = 56)	(*N* = 52)	
Age (years)	58 ± 17	57 ± 13	0.53
Male gender (%)	75	73	0.78
Heart rate (beats/min)	65 ± 7	73 ± 13	<0.001
SBP (mmHg)	120 ± 25	128 ± 21	0.25
DBP (mmHg)	70 ± 10	75 ± 9	0.10
BSA (m^2^)	1.85 ± 0.20	1.82 ± 0.20	0.42
QRS >120 msec	18	0	—
Indexed LVEDV (ml/m^2^)	117 ± 29	55 ± 9	<0.001
Indexed LVESV (ml/m^2^)	81 ± 27	20 ± 4	<0.001
LVEF (%)	31 ± 11	60 ± 19	<0.001
Indexed LAVmax (ml/m^2^)	86 ± 32	29 ± 8	<0.001

BSA: body surface area; DBP: diastolic blood pressure; DCM: dilated cardiomyopathy; LAVmax: left atrium maximal volume; LVEDV: left ventricle end-diastolic volume; LVEF: left ventricle ejection fraction; LVESV: left ventricle end-systolic volume; SBP: systolic blood pressure.

**Table 2:** Comparison of the mitral annulus parameters, measured at mid-systolic frame, between patients with functional mitral regurgitation and control subjects.

	Functional mitral regurgitation	Control subjects	*p*
	(*N* = 56)	(*N* = 52)	
*MA dimensions*			
AP diameter (mm)	3.4 ± 0.5	2.8 ± 0.4	<0.001
ALPM diameter (mm)	4.4 ± 0.5	3.9 ± 0.5	<0.001
Commissural diameter (mm)	4.3 ± 0.5	3.9 ± 0.5	<0.001
MA circumference (cm)	12.9 ± 1.6	11.2 ± 1.2	<0.05
MA area 2D (cm^2^)	12.2 ± 3	8.9 ± 2.1	<0.001
MA area 3D (cm^2^)	12.4 ± 3.1	9.2 ± 2.2	<0.001
ALA (cm^2^)	8.2 ± 1.9	6.2 ± 1.4	<0.001
ALL (cm)	2.8 ± 0.4	2.2 ± 0.3	<0.001
PLA (cm^2^)	7.0 ± 2.06	4.0 ± 1.2	<0.001
PLL (cm)	1.4 ± 0.3	1.0 ± 0.2	<0.001
ALA/PLA ratio	1.2 ± 0.3	1.6 ± 0.5	<0.001

*MA geometry*			
Sphericity index	0.80 ± 0.08	0.72 ± 0.06	<0.001
NPA (°)	157 ± 11	145 ± 10	<0.001
Annular height (mm)	6.2 ± 1.8	6.8 ± 1.5	0.125
Tenting volume (cm^3^)	5.2 ± 1.8	1.9 ± 0.8	<0.001
Tenting area (cm^2^)	2.3 ± 0.7	1.2 ± 0.4	<0.001
Tenting height (mm)	10 ± 2	6 ± 1	<0.001
Angle Ao-AP (°)	145 ± 12	130 ± 13	<0.001

ALA: anterior leaflet area; ALL: anterior leaflet length; angle Ao-AP: angle between aorta and the highest plane of mitral annulus; AP: anteroposterior; ALPM: anterolateral-posteromedial; MA: mitral annulus; PLA: posterior leaflet area; PLL: posterior leaflet length; NPA: nonplanarity angle.

**Table 3:** The dynamic changes of the mitral annulus dimensions and geometry during the cardiac systole.

		Early-systole	Minimal value	Mid-systole	End-systole	Time to minimum (%)
*MA dimensions*						
AP diameter (cm)	FMR	3.3 ± 0.6^*∗*^	3.2 ± 0.6^*∗*^	3.4 ± 0.5^*∗*^	3.6 ± 0.5^*∗*^	26 ± 24^*∗*^
	Controls	2.4 ± 0.3	2.4 ± 0.3	2.8 ± 0.4	2.9 ± 0.3	11 ± 9
ALPM diameter (cm)	FMR	4.2 ± 0.5^*∗*^	4.1 ± 0.5^*∗*^	4.4 ± 0.5^*∗*^	4.4 ± 0.5^*∗*^	26 ± 24^*∗*^
	Controls	3.5 ± 0.4	3.5 ± 0.4	3.9 ± 0.5	3.9 ± 0.5	13 ± 8
Commissural diameter (cm)	FMR	4.1 ± 0.5^*∗*^	4.0 ± 0.5^*∗*^	4.3 ± 0.5^*∗*^	4.3 ± 0.5^*∗*^	28 ± 26^*∗*^
	Controls	3.5 ± 0.4	3.4 ± 0.4	3.9 ± 0.5	3.9 ± 0.4	13 ± 9
MA area 2D (cm^2^)	FMR	11.6 ± 3.1^*∗*^	11.2 ± 3.1^*∗*^	12.2 ± 3.1^*∗*^	12.8 ± 3.8^*∗*^	20 ± 19^*∗*^
	Controls	7.4 ± 1.7	7.2 ± 1.6	8.9 ± 2.1	9.6 ± 2.0	11 ± 6
MA area 3D (cm^2^)	FMR	11.8 ± 3.2^*∗*^	11.4 ± 3.11^*∗*^	12.4 ± 3.1^*∗*^	13.0 ± 3.2^*∗*^	20 ± 18^*∗*^
	Controls	7.7 ± 1.8	7.4 ± 1.7	9.2 ± 2.2	9.8 ± 2.0	11 ± 6
MA circumference (cm)	FMR	12.6 ± 1.6^*∗*^	12.3 ± 1.6^*∗*^	12.9 ± 1.6^*∗*^	13.1 ± 1.6^*∗*^	21 ± 20^*∗*^
	Controls	10.3 ± 1.1	10.2 ± 1.1	11.2 ± 1.3	11.6 ± 1.2	11 ± 6

*MA geometry*						
Sphericity index	FMR	0.8 ± 0.09^*∗*^	0.75 ± 0.08^*∗*^	0.8 ± 0.08^*∗*^	0.8 ± 0.08^*∗*^	41 ± 28^*∗*^
	Controls	0.7 ± 0.07	0.65 ± 0.06	0.7 ± 0.06	0.75 ± 0.07	20 ± 18
NPA (°)	FMR	162 ± 10^*∗*^	153 ± 10^*∗*^	157 ± 11^*∗*^	159 ± 10^*∗*^	48 ± 27
	Controls	152 ± 14	140 ± 9	145 ± 10	146 ± 8	55 ± 31
Annular height (mm)	FMR	5.2 ± 0.1^*∗*^	4.7 ± 0.1^*∗*^	5.9 ± 0.2^*∗*^	5.7 ± 0.2^*∗*^	43 ± 37^*∗*^
	Control	5.9 ± 0.1	5.6 ± 0.1	6.9 ± 0.2	6.8 ± 0.1	27 ± 33
Tenting volume (cm^3^)	FMR	6.4 ± 2.3^*∗*^	4.6 ± 1.7^*∗*^	5.2 ± 1.8^*∗*^	5.3 ± 2.1^*∗*^	78 ± 18
	Control	2.9 ± 1.2	1.4 ± 0.7	1.9 ± 0.8	2.1 ± 1.0	78 ± 14
Tenting area (cm^2^)	FMR	2.3 ± 0.8^*∗*^	2.1 ± 0.7^*∗*^	2.4 ± 0.7^*∗*^	2.4 ± 0.8^*∗*^	69 ± 28
	Control	1.5 ± 0.5	1.0 ± 0.3	1.2 ± 0.3	1.4 ± 0.5	70 ± 24
Tenting height (mm)	FMR	11.5 ± 2.3^*∗*^	8.7 ± 2.1^*∗*^	10 ± 2.2^*∗*^	9.3 ± 2.6^*∗*^	84 ± 19
	Control	8.4 ± 1.7	4.5 ± 1.5	6.3 ± 1.5	6.1 ± 2.0	79 ± 14
Angle Ao-AP (°)	FMR	147 ± 13^*∗*^	142 ± 12^*∗*^	145 ± 12^*∗*^	149 ± 12^*∗*^	39 ± 28^*∗*^
	Control	137 ± 11	123 ± 11	130 ± 13	124 ± 11	24 ± 14

Angle Ao-AP: angle between aorta and the highest plane of mitral annulus; AP: anteroposterior; ALPM: anterolateral-posteromedial; MA: mitral annulus; NPA: nonplanarity angle. ^*∗*^Statistical difference with *p* < 0.01.

**Table 4:** Fractional changes of the mitral annulus parameters during the cardiac systole.

	Functional mitral regurgitation	Control subjects	*p*
	(*N* = 56)	(*N* = 52)	
MA area fraction (%)	14.6 ± 5.0	27.6 ± 4.6	<0.001
AP diameter change (%)	10 ± 6	19 ± 7	<0.001
ALPM diameter change (%)	7 ± 4	12 ± 4	<0.001
MA displacement (mm)	4.8 ± 2.9	10.2 ± 1.7	<0.001
Displacement velocity (mm/sec)	30 ± 13	50 ± 9	<0.001

AP: anteroposterior; ALPM: anterolateral-posteromedial; MA: mitral annulus.

**Table 5:** Comparison between patients with trivial/mild, moderate, and severe FMR.

Parameters	Trivial/mild FMR (*N* = 17)	Moderate FMR (*N* = 25)	Severe FMR (*N* = 14)	One-way ANOVA between MR severity groups	P (mild vs. moderate FMR)	P (moderate vs. severe FMR)
Indexed LVEDV (ml/m^2^)	102 ± 24	121 ± 31	130 ± 28	0.002	<0.05	0.80
Indexed LVESV (ml/m^2^)	65 ± 21	88 ± 30	90 ± 17	0.07	0.10	0.36
LVEF (%)	37 ± 10	28 ± 10	29 ± 9	0.02	<0.01	0.69
Indexed LAVmax (ml/m^2^)	38 ± 12.5	45 ± 16	59 ± 12	0.006	0.15	<0.05
Total LAEF (%)	40 ± 18	29 ± 14	23 ± 13	0.02	<0.05	0.20
True LAEF (%)	20 ± 10	18 ± 10	13 ± 9	0.22	0.64	0.13
MA AP diameter (cm)	3.1 ± 0.4	3.4 ± 0.4	3.9 ± 0.5	<0.001	<0.05	<0.001
MA ALPM diameter (cm)	4.1 ± 0.5	4.4 ± 0.46	4.6 ± 0.5	0.007	<0.05	0.15
MA circumference (cm)	11.8 ± 1.4	13.0 ± 2.2	14.0 ± 1.6	<0.001	<0.01	<0.05
MA area 3D (cm^2^)	10.4 ± 2.5	12.4 ± 2.3	14.8 ± 3.4	<0.001	<0.05	<0.01
MA area fractional change (%)	18.6 ± 5.3	14 ± 4.3	10.6 ± 3.0	<0.001	<0.01	<0.05
MA AP diameter change (%)	11.6 ± 5.7	10 ± 5.7	8.0 ± 6	0.22	0.37	0.14
MA ALPM diameter change (%)	9.6 ± 4.5	7.3 ± 4.0	5.4 ± 2.8	0.02	0.08	0.29
MA displacement (mm)	6.3 ± 2.6	3.9 ± 2.1	4.6 ± 3.8	0.03	<0.01	0.44

AP: anteroposterior; ALPM: anterolateral-posteromedial; LAEF: left atrium emptying fraction; LAVmax: left atrium maximal volume; LVEDV: left ventricle end-diastolic volume; LVESV: left ventricular end-systolic volume; LVEF: left ventricular ejection fraction; MA: mitral annulus.

## Data Availability

All study information is available upon request from the corresponding author. For this purpose, the corresponding and first author of the paper may be contacted via the e-mail sorinamihaila1981@gmail.com.

## Additional Points

Functional mitral regurgitation is a common and morbid complication in patients with ischemic or nonischemic dilated cardiomyopathy, and its management remains challenging. It is known that the mitral valve apparatus presents altered size, geometry, and dynamics in patients with functional mitral regurgitation, which is essential information in order to design new devices and to properly select patients to treat this disease. In patients with functional mitral regurgitation, the mitral annulus has increased size and sphericity and decreased height during the entire cardiac systole, coupled with a significant remodeling of the mitral valve leaflets. By using three-dimensional echocardiography, our study showed that the mitral annulus from patients with functional mitral regurgitation is larger, more spherical, and flatter during the entire cardiac systole. Moreover, the contraction of the mitral annulus is decreased and delayed in time and positively related to the functional mitral regurgitation severity. Finally, the contractile dysfunction of the mitral annulus related not only to the left ventricular systolic dysfunction but also to the left atrial pump dysfunction.
